# Neurological manifestations as the predictors of severity and mortality in hospitalized individuals with COVID-19: a multicenter prospective clinical study

**DOI:** 10.1186/s12883-021-02152-5

**Published:** 2021-03-16

**Authors:** Man Amanat, Nima Rezaei, Mehrdad Roozbeh, Maziar Shojaei, Abbas Tafakhori, Anahita Zoghi, Ilad Alavi Darazam, Mona Salehi, Ehsan Karimialavijeh, Behnam Safarpour Lima, Amir Garakani, Alexander Vaccaro, Mahtab Ramezani

**Affiliations:** 1 Faculty of Medicine, Students’ Scientific Research Center, Tehran University of Medical Sciences, Tehran, Iran; 2 Department of Immunology, School of Medicine, Tehran University of Medical Sciences, Tehran, Iran; 3 Network of Immunity in Infection, Malignancy and Autoimmunity (NIIMA), Universal Scientific Education and Research Network (USERN), Tehran, Iran; 4 Research Center for Immunodeficiencies, Children’s Medical Center, Tehran University of Medical Sciences, Tehran, Iran; 5 Department of Neurology, Brain Mapping Research Center, Shahid Beheshti University of Medical Sciences, Tehran, Iran; 6 Department of Neurology, Shahid Beheshti University of Medical Sciences, Tehran, Iran; 7 Iranian Center of Neurological Research, Neuroscience Institute, Tehran University of Medical Sciences, Tehran, Iran; 8 Department of Infectious disease and Tropical Medicine, Shahid Beheshti University of Medical Sciences, Tehran, Iran; 9 Department of Emergency Medicine, Tehran University of Medical Sciences, Tehran, Iran; 10 Department of Psychiatry, Yale School of Medicine, New Haven, CT USA; 11 Department of Psychiatry, Icahn School of Medicine at Mount Sinai, New York, NY USA; 12 Department of Orthopedics and Neurosurgery, Rothman Institute, Thomas Jefferson University, Philadelphia, PA USA; 13 Department of Neurology, Skull Base Research Center, Shahid Beheshti University of Medical Sciences, Tehran, Iran

**Keywords:** SARS-CoV-2, Anosmia, Headache, Neurology, Seizure, Stroke

## Abstract

**Backgrounds:**

The reports of neurological symptoms are increasing in cases with coronavirus disease 2019 (COVID-19). This multi-center prospective study was conducted to determine the incidence of neurological manifestations in hospitalized cases with COVID-19 and assess these symptoms as the predictors of severity and death.

**Methods:**

Hospitalized males and females with COVID-19 who aged over 18 years were included in the study. They were examined by two neurologists at the time of admission. All survived cases were followed for 8 weeks after discharge and 16 weeks if their symptoms had no improvements.

**Results:**

We included 873 participants. Of eligible cases, 122 individuals (13.97%) died during hospitalization. The most common non-neurological manifestations were fever (81.1%), cough (76.1%), fatigue (36.1%), and shortness of breath (27.6%). Aging, male gender, co-morbidity, smoking, hemoptysis, chest tightness, and shortness of breath were associated with increased odds of severe cases and/or mortality. There were 561 (64.3%) cases with smell and taste dysfunctions (hyposmia: 58.6%; anosmia: 41.4%; dysguesia: 100%). They were more common among females (69.7%) and non-smokers (66.7%). Hyposmia/anosmia and dysgeusia were found to be associated with reduced odds of severe cases and mortality. Myalgia (24.8%), headaches (12.6%), and dizziness (11.9%) were other common neurological symptoms. Headaches had negative correlation with severity and death due to COVID-19 but myalgia and dizziness were not associated. The cerebrovascular events (*n* = 10) and status epilepticus (*n* = 1) were other neurological findings. The partial or full recovery of smell and taste dysfunctions was found in 95.2% after 8 weeks and 97.3% after 16 weeks. The parosmia (30.9%) and phantosmia (9.0%) were also reported during 8 weeks of follow-up. Five cases with mild headaches and 5 cases with myalgia were reported after 16 weeks of discharge. The demyelinating myelitis (*n* = 1) and Guillain-Barré syndrome (*n =* 1) were also found during follow-up.

**Conclusion:**

Neurological symptoms were found to be prevalent among individuals with COVID-19 disease and should not be under-estimated during the current pandemic outbreak.

## Introduction

The novel virus from the coronaviridae family termed as severe acute respiratory syndrome coronavirus 2 (SARS-CoV-2) was first discovered in December, 2019 after many pneumonia cases with unknown cause were identified in Wuhan, China [1]. The SARS-CoV-2 is a β-coronavirus which is an enveloped virus with helical nucleocapsid and non-segmented positive ribonucleic acid that can infect mammals [2–5]. The World Health Organization (WHO) declared coronavirus disease 2019 (COVID-19) as a pandemic outbreak on March 11th, 2020. Over 80 million confirmed cases and about 2 million deaths due to COVID-19 were recorded until the end of 2020 [6].

Several non-respiratory symptoms have been reported in individuals with COVID-19. The reports of neurological features are increasing but few large sample-sized studies reported the incidence of neurological disorders in people with COVID-19. Myalgia, headaches, and loss of smell (anosmia) and taste (dysgeusia) sensations are common neurological findings of the disease [7–9]. Recent studies showed that SARS-CoV-2 could manifest as life-threatening neurological conditions including ischemic stroke, subarachnoid hemorrhage, status epilepticus, and acute demyelinating encephalomyelitis [10–13]. Neurological symptoms were also reported in epidemic outbreaks of other coronaviruses including SARS-CoV and the Middle East respiratory syndrome (MERS)-CoV [14, 15]. The direct invasion of coronaviruses to the nervous system and over-production of pro-inflammatory cytokines could be the plausible underlying mechanisms [16]. This study aimed to determine the incidence of neurological manifestations in hospitalized cases with COVID-19 and assess these symptoms as predictors of severity and death.

## Methods

### Study design and participants

This was a prospective hospital-based cohort study conducted in Loghman, Imam Hossein, and Imam Khomeini hospitals, the three major referral hospitals in Tehran province, Iran. Males and females aged above 18 years who were diagnosed with COVID-19 based on WHO recommendations [17] were included in the study. The sterile nasopharyngeal swabs were inserted in one nostril of each participant. The collected specimens were placed into tubes containing viral transport medium; stored at 4^o^ C to 8^o^ C and were sent to laboratories within 12 h. The positive results of real-time reverse-transcription polymerase chain reaction (RT-PCR) assay using a SARS-CoV-2 nucleic acid detection kit (PCR Fluorescence Probing of Sansure Biotech, Changsha, China) could confirm COVID-19. The exclusion criteria were the presence of other respiratory infections (e.g. tuberculosis), loss of competence and no access to surrogates, and withdrawal of consent.

The ethics committee of Shahid Beheshti University of Medical Sciences approved the study. The protocol was explained to all participants and if they lacked decision-making capacity, their legal surrogates were informed about the study. The printed protocol was also given. It was explained that participation in the study was optional and the identity information would remain confidential and not published. Written informed consent was obtained before the initiation of the study.

### Data collection

All eligible inpatients were interviewed and the demographic characteristics including gender, age, smoking status, and co-morbidities were recorded. Participants were examined and chest computed tomography (CT) was performed in all cases. The laboratory tests included cell blood count, quantitative C-reactive protein (CRP), lactate dehydrogenase (LDH), and procalcitonin. The severity of COVID-19 was determined using American Thoracic Society recommendations for community-acquired pneumonia [18].

To assess neurological manifestations, two neurologists with at least 5 years of experience took a detailed history and examined all participants. Any disagreement was resolved by discussion with each other or consultation with another neurologist. Headaches were known to be associated with COVID-19 if they fulfilled the criteria for ‘Headache attributed to systemic viral infection’ according to the International Classification of Headache Disorders [19]. Brain CT and/or magnetic resonance imaging (MRI), electroencephalography (EEG), electromyography and nerve conduction velocity (EMG-NCV), as well as cerebrospinal fluid (CSF) analysis were conducted if clinically indicated. Smell and taste dysfunctions were assessed in cases using a self-reporting tool. The National Institute of Health stroke scale (NIHSS) was used to evaluate the severity of ischemic stroke. The NIHSS was designed to objectively quantify neurological impairments in stroke and consisted of 11 items [20]. Each item can be scored from 0 to 4 and the total possible scores ranged from 0 to 42. Neurological impairments can be divided into four categories using NIHSS: a. mild (0–4), b. moderate (5–15), c. Moderate to severe (16–20), and d. severe (21–42). The intracerebral hemorrhage (ICH) score was also calculated in participants with ICH. This is a clinical grading scale using Glasgow Coma Scale, age, the presence of infratentorial origin, ICH volume, and the presence of intraventricular hemorrhage to predict the ICH mortality [21]. The total score ranged from 0 to 6.

Most included cases were followed for 8 weeks after discharge from hospital to assess the course of their neurological manifestations. If their neurological symptoms were persistent, they were followed for further 8 weeks. Cellphone and telephone numbers of each participant were recorded and they were interviewed via phone once per 4 weeks by a neurologist. If cases reported any new-onset neurological complaints, they would be asked to visit the hospital for further assessments. A phone number was also given to participants so physical complaints could be reported as soon as possible. Participants were asked to go to the emergency department if serious events occurred.

### Statistical analysis

Continuous variables were presented as means with standard deviation (SD) and compared between groups using independent sample t test. Categorical variables were described as numbers with percentages and compared between groups using Pearson’s chi-squared test. To more rigorously assess the predictors of severity and death due to COVID-19, odds ratio (OR) with 95% confidence interval (CI) was calculated using univariate and multivariate logistic regression.. The calculated ORs of neurological manifestations were adjusted for gender, age, smoking status, and co-morbidity. All analyses were conducted using the IBM SPSS Software version 25.0 (SPSS Inc., Chicago, IL). Two sided significance testing was performed and *p*-values < 0.05 were considered significant.

## Results

### Participants

**Fig. 1:**
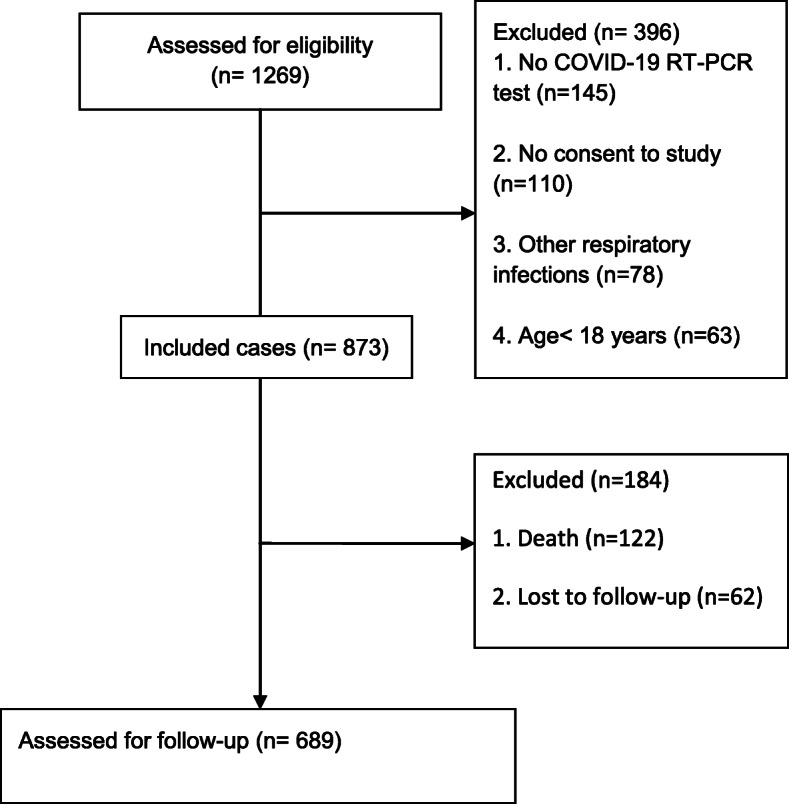
The study diagram

The study was initiated on April 7, 2020 when the clinical characteristics of the first hospitalized participant were assessed. The follow-up of survived cases lasted until November 18, 2020. Primary screening was performed on 1269 inpatients and 873 cases were included in the study (Fig. 1). Of eligible participants, 122 individuals died during hospitalization (case fatality rate: 13.97%) and 751 cases were discharged in 11.41 ± 6.20 (mean ± SD) days. There were 62 participants who were lost to follow-up and 689 individuals completed the study.

### Non-neurological characteristics

The demographic clinical features of included cases during hospitalization were presented in Table 1. There were 551 males (63.7%) and 317 females (36.3%). The mean ± SD age of participants was 60.71 ± 18.14 years and 459 individuals (52.6%) aged over 60 years. The most common non-neurological manifestations were fever (708/873, 81.1%), cough (664/873, 76.1%), fatigue (315/873, 36.1%), and shortness of breath (241/873, 27.6%). Our analysis showed that aging (severity: OR: 1.020, 95%CI: 1.012 to 1.028; death: OR: 1.08, 95%CI: 1.06 to 1.99), male gender (severity: OR: 1.38, 95%CI: 1.03 to 1.86), presence of co-morbidity (OR: 1.43, 95%CI: 1.11 to 1.98; death: OR: 2.81, 95%CI: 1.79 to 4.42), smoking (severity: OR: 1.62, 95%CI: 1.11 to 2.34; death: 2.69, 95%CI: 1.73 to 4.18), hemoptysis (severity: OR: 3.55, 95%CI: 2.01 to 6.28; death: OR: 2.30, 95%CI: 1.21 to 4.36), chest tightness (severity: OR: 3.23, 95%CI: 2.09 to 5.00; death: OR: 2.63, 95%CI: 1.60 to 4.32), and shortness of breath (severity: OR: 2.51, 95%CI: 1.84 to 3.41; death: OR: 9.61, 95%CI: 6.25 to 14.92) were associated with increased odds of severity and/or death in participants.

**Table 1:** Demographic characteristics and non-neurological clinical manifestations of participants

Clinical features	Total	Disease severity			Death outcome		***P***-value
	***N*** = 873	Non-severe (***N*** = 574)	Severe (***N*** = 299)	***P***-value	No (***N*** = 751)	Yes (***N*** = 122)	
Age (mean ± SD)	60.71 ± 18.14	58.57 ± 17.95	64.82 ± 17.95	**0.00**	58.12 ± 17.59	76.68 ± 12.46	**0.00**
≤60 years	414 (47.4)	296 (51.6)	118 (39.5)		400 (53.3)	14 (11.5)	
> 60 years	459 (52.6)	278 (48.4)	181 (60.5)	**0.00**	351 (46.7)	108 (88.5)	**0.00**
Gender							
Female	317 (36.3)	223 (38.9)	94 (31.4)		280 (37.3)	37 (30.3)	
Male	556 (63.7)	351 (61.1)	205 (68.6)	**0.03**	471 (62.7)	85 (69.7)	0.16
Smoking	137 (15.7)	77 (13.4)	60 (20.1)	**0.01**	101 (13.4)	36 (29.5)	**0.00**
Co-morbidities	512 (58.6)	318 (55.4)	194 (64.9)	**0.01**	417 (55.5)	95 (77.9)	**0.00**
Fever	708 (81.1)	472 (82.2)	236 (78.9)	0.23	614 (81.8)	94 (77.0)	0.21
Cough	664 (76.1)	429 (74.7)	235 (78.6)	0.21	572 (76.2)	92 (75.4)	0.91
Fatigue	315 (36.1)	205 (35.7)	110 (36.8)	0.76	264 (35.2)	51 (41.8)	0.16
Shortness of breath	241 (27.6)	121 (21.1)	120 (40.1)	**0.00**	154 (20.5)	87 (71.3)	**0.00**
Sputum production	156 (17.9)	94 (16.4)	62 (20.7)	0.11	133 (17.7)	23 (18.9)	0.80
Chills	101 (11.6)	72 (12.5)	29 (9.7)	0.22	88 (11.7)	13 (10.7)	0.88
Sore throat	97 (11.1)	58 (10.1)	39 (13.0)	0.21	84 (11.2)	13 (10.7)	1.00
Chest tightness	96 (11)	39 (6.8)	57 (19.1)	**0.00**	70 (9.3)	26 (21.3)	**0.00**
Nausea	94 (10.8)	64 (11.1)	30 (10.0)	0.65	81 (10.8)	13 (10.7)	1.00
Vomiting	36 (4.1)	26 (4.5)	10 (3.3)	0.48	32 (4.3)	4 (3.3)	0.80
Diarrhea	32 (3.7)	23 (4.0)	9 (3.0)	0.57	28 (3.7)	3 (2.5)	1.00
Hemoptysis	54 (6.2)	20 (3.5)	34 (11.4)	**0.00**	40 (5.3)	14 (11.5)	**0.01**
Rhinorrhea	49 (5.6)	26 (4.5)	23 (7.7)	0.06	40 (5.3)	9 (7.4)	0.39
Nasal congestion	24 (2.7)	15 (2.6)	9 (3.0)	0.83	23 (3.1)	1 (0.8)	0.23
Conjunctival congestion	4 (0.5)	2 (0.3)	2 (0.7)	0.61	4 (0.5)	0 (0)	1.00

Categorical variables are presented as numbers (percentages); SD: standard deviation

The most common laboratory abnormalities were elevated CRP (542/873, 61.2%), lymphocytopenia (434/873, 49.7%), elevated LDH (316/873, 35.7%), and neutrophilia (291/873, 33.3%) (Table 2). The statistical analysis showed that the elevated procalcitonin (severity: OR: 2.26, 95%CI: 1.35 to 3.78; death: OR: 5.40; 95%CI: 3.12 to 9.34), elevated LDH (severity: OR: 1.99, 95%CI: 1.49 to 2.65; death: OR: 10.63; 95%CI: 6.53 to 17.24), neutrophilia (severity: OR: 1.68, 95%CI: 1.26 to 2.26; death: OR: 3.10; 95%CI: 2.09 to 4.58), elevated CRP (severity: OR: 1.46, 95%CI: 1.09 to 1.96; death: OR: 6.17; 95%CI: 3.40 to 11.11), leukocytosis (death: OR: 1.62, 95%CI: 1.04 to 2.53), and lymphocytopenia (severity: OR: 1.36, 95%CI: 1.03 to 1.81) were associated with increased odds of severity and/or death due to COVID-19.

**Table 2:** The laboratory data of participants

Laboratory data	Total	Disease severity			Death outcome		***P***-value
	***N =*** 873	Non-severe (***N =*** 574)	Severe (***N =*** 299)	***P***-value	No (***N =*** 751)	Yes (***N =*** 122)	
Leukopenia < 3500/mm^3^	39 (4.5)	20 (3.5)	19 (6.4)	0.06	30 (4.0)	9 (7.4)	0.09
Leukocytosis > 11,000/mm^3^	167 (19.1)	108 (18.8)	59 (19.7)	0.79	135 (18)	32 (26.2)	**0.03**
Neutropenia < 1500/mm^3^	17 (1.9)	12 (2.1)	5 (1.7)	0.80	14 (1.9)	3 (2.5)	0.72
Neutrophilia > 8000/mm^3^	291 (33.3)	168 (29.3)	123 (41.1)	**0.00**	222 (29.6)	69 (56.6)	**0.00**
Lymphocytopenia < 1000/mm^3^	434 (49.7)	270 (47.0)	164 (54.8)	**0.03**	364 (48.5)	70 (57.4)	0.08
Lymphocytosis > 4000/mm^3^	17 (1.9)	11 (1.9)	6 (2.0)	1.00	15 (2.0)	2 (1.6)	1.00
Thrombocytopenia < 100,000/mm^3^	65 (7.4)	39 (6.8)	26 (8.7)	0.34	56 (7.5)	9 (7.4)	1.00
Thrombocytosis > 450,000/mm^3^	11 (1.3)	7 (1.2)	4 (1.3)	1.00	11 (1.5)	0 (0)	0.38
Elevated LDH > 245 U/liter	316 (36.2)	176 (30.7)	140 (46.8)	**0.00**	217 (28.9)	99 (81.1)	**0.00**
Elevated CRP > 10 mg/liter	542 (62.1)	339 (59.1)	203 (67.9)	**0.01**	433 (57.7)	109 (89.3)	**0.00**
Elevated Procalcitonin > 0.5 ng/ml	63 (7.2)	30 (5.2)	33 (11.0)	**0.00**	37 (4.9)	26 (21.3)	**0.00**

Categorical variables are presented as numbers (percentages); *LDH* lactate dehydrogenase, *CRP* C-reactive protein

### Neurological manifestations

**Fig. 2:**
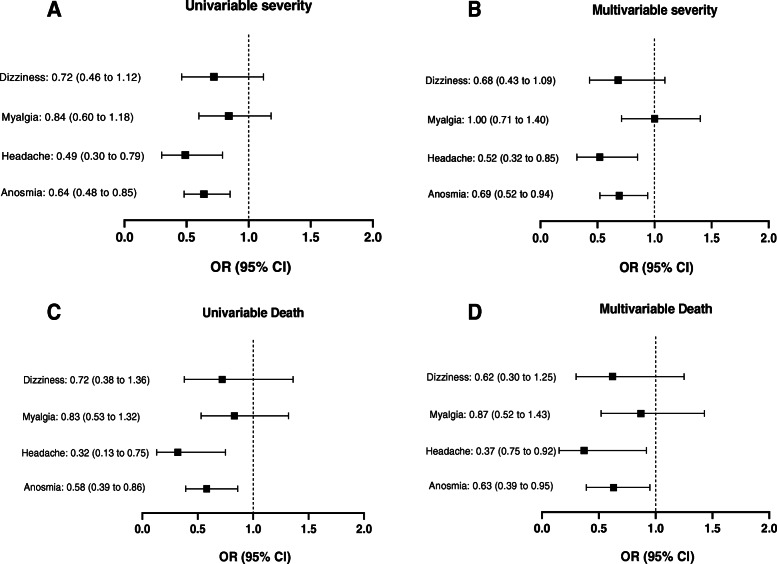
The prediction of severity and death due to COVID-19 by neurological manifestations

Smell and taste dysfunctions were reported in 561 (64.3%) included cases at the time of admission (hyposmia: 329/561 or 58.6%; anosmia: 232/561 or 41.4%; dysgeusia: 561/561 or 100%). They were more common among females (69.7%) than males (OR: 1.46, 95%CI: 1.08 to 1.96) and also more frequent among non-smokers (66.7%) than smokers (OR: 1.91, 95%CI: 1.32 to 2.77). Co-morbidities were more frequent among cases with normal smell and taste sensations (OR: 1.42, 95%CI: 1.07 to 1.89). There were 28 cases with rhinorrhea and 17 cases with nasal congestion who reported loss of smell sensation. Of 561 participants, 142 individuals (25.3%) reported these symptoms as the first clinical manifestation. Hyposmia/anosmia and dysgeusia were found to be associated with reduced odds of severe cases (OR: 0.69, 95%CI: 0.52 to 0.94) and death (OR: 0.63, 95%CI: 0.39 to 0.95) after adjustment for gender, age, smoking status, co-morbidity, rhinorrhea, and nasal congestion (Table 3, Fig. 2).

**Table 3:** The common neurological symptoms among participants

Neurological symptoms	Total	Disease severity			Death outcome		***P***-value
	***N =*** 873	Non-severe (***N =*** 574)	Severe (***N =*** 299)	***P***-value	No (***N =*** 751)	Yes (***N =*** 122)	
Smell and taste dysfunctions	561 (64.3)	389 (67.8)	172 (57.5)	**0.00**	496 (66.0)	65 (53.3)	**0.00**
Myalgia	217 (24.9)	142 (24.7)	75 (25.1)	0.93	190 (25.3)	27 (22.1)	0.50
Headaches	110 (12.6)	86 (15.0)	24 (8.0)	**0.00**	104 (13.8)	6 (4.9)	**0.00**
Dizziness	104 (11.9)	75 (13.1)	29 (9.7)	0.16	94 (12.5)	10 (8.2)	0.37

Categorical variables are presented as numbers (percentages)

Myalgia (217/873 or 24.9%), headaches (110/873 or 12.6%), and dizziness (104/873 or 11.9%) were other common neurological symptoms. The incidence rates of these conditions were not significantly different among genders. Of 110 individuals with headaches, 71 cases (64.5%) had migraine-like episodes; 37 participants (33.6%) had tension-like headaches; and 2 cases (1.9%) had only cough-related headaches. Headaches had negative correlation with severity (adjusted OR: 0.52, 95%CI: 0.32 to 0.85) and death due to COVID-19 (adjusted OR: 0.37, 95%CI: 0.75 to 0.92) but myalgia and dizziness were not associated (Table 3, Fig. 2).

There were 10 cases (1.26%) with COVID-19 who presented cerebrovascular events (Table 4). The mean age was 63.40 years (SD: 20.55). Brain CT scans were performed in all cases and ischemic stroke was found in 7 patients (70%) and 3 cases (30%) were diagnosed with ICH. Of cases with ischemic stroke, mild impairment was observed in 1 case with NIHSS < 5. We found 3 cases with moderate (NIHSS: 5–15), 1 case with moderate to severe (NIHSS: 16–20), and 2 cases with severe (NIHSS > 21) neurological impairments. The ICH score of 4 was calculated in 2 cases and the score of 3 was calculated in 1 patient with ICH. Fever (*n* = 10), cough (*n* = 8), fatigue (*n* = 7), myalgia (*n* = 3), and headaches (*n =* 1) were reported in 10.7 ± 2.91 days prior to cerebrovascular events but none were severe COVID-19 cases. Six patients (60%) died in 4.15 ± 2.23 days after hospitalization.

**Table 4:** Characteristic of participants with cerebrovascular events

Age (years)	Gender	Co-morbidities	Type of event	Localization	NIHSS/ICH score	Days from first symptom to event	Death
75	Male	Yes	Ischemic stroke	Superior cerebellar artery embolism	4	11	No
18	Female	No	ICH	Left putamen hemorrhage	4	11	Yes
63	Male	Yes	Ischemic stroke	Left Middle cerebral artery plaque	24	13	Yes
65	Male	Yes	Ischemic stroke	Left Middle cerebral artery plaque	15	8	No
83	Female	Yes	ICH	Right thalamus hemorrhage	3	8	Yes
72	Male	Yes	Ischemic stroke	Right Middle cerebral artery plaque	9	7	No
85	Male	Yes	Ischemic stroke	Left Middle cerebral artery plaque	23	8	Yes
38	Female	Yes	ICH	Left putamen hemorrhage	4	13	Yes
68	Female	Yes	Ischemic stroke	Right Middle cerebral artery plaque	17	12	Yes
67	Male	No	Ischemic stroke	Right Middle cerebral artery plaque	10	16	No

**Fig. 3:**
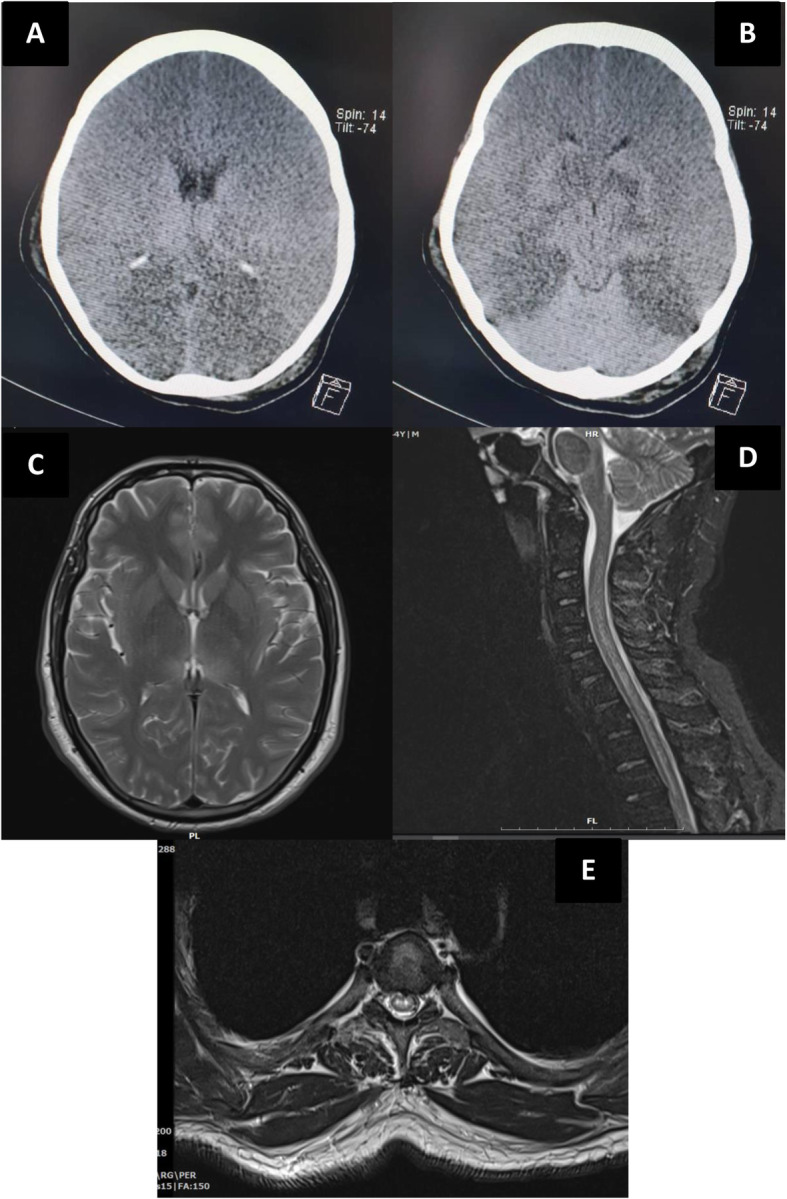
Brain imaging of individuals with status epilepticus (**a**, **b**) and demyelinating myelitis (**c**, **d**, **e**)

One previously healthy 28 year-old-female was referred to Loghman hospital with status epilepticus as the sole clinical manifestation. No history of physical disorders, head trauma, drug use, or recent respiratory symptoms was reported from parents. Physical examination revealed bilateral mydriatic and non-reactive pupils with downward gaze. The EEG showed moderate to severe diffuse slowing and generalized rhythmic delta activity. She was intubated and received intravenous levetiracetam (3000 mg) and generalized tonic-clonic seizure occurred within 2 h of drug administration. Intravenous midazolam was, then, added. No other convulsive seizures were developed during hospitalization. Bilateral ground glass opacities were observed in chest CT and the RT-PCR test for COVID-19 was positive. Cerebrospinal (CSF) analysis showed clear appearance with no cells, protein: 79 mg/dl (normal: 15–45), glucose: 135 mg/dl (normal: 45–80), and opening pressure: 22 cmH_2_O (normal: 6–25). The PCR panel of CSF was negative for herpes simplex and no bacterial or fungal organisms were detected. Other laboratory data showed thrombocytopenia (*n* = 43,000) at the time of admission but other tests were in normal limits (leukocytes: 5.1 × 10^3^/μL, Na: 142, K: 4.4, Ca: 9.4, Albumin: 3.4, and Mg: 2.1). Urine toxicology analysis for methanol, methadone, benzodiazepines, cannabinoids, methamphetamine, tramadol, and opiates were negative. The primary brain CT scans showed no specific abnormalities but the top of basilar infarction was observed after ten days of hospitalization (Fig. 3 a, b). The patient died 16 days after admission.

### Follow-up

**Fig. 4:**
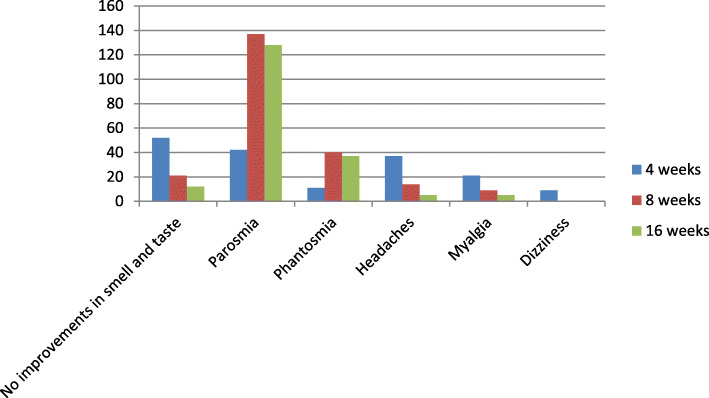
Number of participants with neurological symptoms during follow-up

There were 442 individuals with hyposmia/anosmia and dysgeusia who survived from COVID-19 and completed the study. It was found that after 4 weeks of discharge, 209 cases (47.3%) fully recovered from these conditions; 181 cases (40.9%) had partial improvements; and 52 cases (11.8%) reported no changes in their smell and taste dysfunctions. The full recovery was reported in 302 individuals (68.3%) after 8 weeks of discharge and partial improvements were found among 119 participants (26.9%). Twenty one cases (4.8%) with hyposmia (*n* = 15) and anosmia (*n* = 6) reported no changes (Fig. 4). Intermittent parosmia (distortion of odor perception when an odor is present) was reported in 42 cases (9.5%) after 4 weeks and 137 cases (30.9%) after 8 weeks of discharge. Intermittent phantosmia (odor perception in the absence of odor stimulus) was also reported in 11 cases (2.5%) after 4 weeks and 40 cases (9.0%) after 8 weeks of discharge (Fig. 4). These events occurred in 86 cases (48.6%) with recovered hyposmia/anosmia, 83 cases (46.9%) with partial improvements, and eight case (4.5%) with no smell and taste improvements.

Headaches were reported among 37 of 689 individuals after 4 weeks of discharge (5.3%) (28 cases with migraine-like and 9 cases with tension-like headaches) and 14 cases (2.0%) after 8 weeks (11 cases with migraine-like and 3 cases with tension-like headaches). These individuals had no prior history of headaches (e.g. migraine). The severity was mild to moderate and headaches were well-controlled with simple analgesics. No functional impairments were reported among affected individuals due to headaches. There were 21 participants (3.0%) after 4 weeks and 9 cases (1.3%) after 8 weeks of discharge with myalgia. Dizziness was also reported among 9 individuals after 4 weeks that was recovered after 8 weeks of discharge (Fig. 4).

The follow-up was continued in participants with parosmia, phantosmia, no improvements in smell and taste, headaches, and myalgia (Fig. 4). The full recovery was reported in 9 cases (6.6%) with parosmia, 3 cases (5.0%) with phantosmia, 2 cases (9.5%) with no prior changes in smell and taste sensations, 9 cases (64.3%) with headaches, and 4 cases with myalgia (44.4%) after 8 further weeks of follow-up (16 weeks after discharge). Partial improvements were reported in 64 cases (46.7%) with parosmia, 12 cases with phantosmia (30.0%), and 7 cases (33.3%) with no prior changes in smell and taste sensations after 16 weeks of discharge. The remaining cases with headaches had mild migraine-like episodes.

#### Demyelinating myelitis

One 43-year-old-male visited Loghman hospital after 16 days of prior admission with weakness, paresthesia, and paresis of upper and lower limbs. The patient also developed low abdominal pain with urinary retention. No history of similar symptoms and neurological disorders were reported. The initial manifestations due to COVID-19 were fever, fatigue, myalgia, and headaches that were recovered after discharge. Neurological examinations showed reduced forces of upper (4 / 5) and lower limbs (2 / 5). The deep tendon reflexes of all extremities were normal (1+) and Babinski’s sign was present. The position, vibration, and light tough sensations were impaired in lower limbs. The laboratory data including leukocyte count, CRP, and LDH were normal. The CSF analysis showed clear appearance with no cells, protein: 60 mg/dl, glucose: 43 mg/dl, and opening pressure: 18 cmH_2_O. The PCR panel of CSF was negative for herpes simplex and no bacterial or fungal organisms were detected. The CSF was assessed to detect anti-N-methyl-D-aspartate (NMDA) antibody, oligoclonal band, aquaporin-4 receptor (AQP-4), and myelin oligodendrocyte glycoprotein (MOG) antibodies which were negative. Serological testing for the anti-nuclear antibody, anti-phospholipid antibodies, neuromyelitis optica, anti-NMDA, AQP-4, and MOG antibodies was also negative. The brain and spinal cord MRI showed bilateral thalamic hypersignal lesions (Fig. 3c) with longitudinally extensive transverse myelitis at cervical and thoracic cord levels (Fig. 3 d, e). The patient was diagnosed with post-infectious demyelinating myelitis and managed with intravenous immunoglobulin (IVIG) for 7 days. Our patient was discharged from hospital after 16 days with significant improvements in forces of upper (5 / 5) and lower limbs (4 / 5).

#### Guillain-Barré syndrome (GBS)

A 46-year-old-female visited Imam Hossein hospital after 21 days of prior admission with weakness and paresthesia of upper and lower limbs as well as bilateral facial paralysis. The initial symptoms due to COVID-19 were fever and dry cough that were recovered after discharge. Neurological examination showed decreased forces of upper (4 / 5) and lower limbs (3 / 5) with mild bilateral peripheral facial nerve palsy. The deep tendon reflexes of all extremities were absent. The CSF analysis showed no cells, protein: 93 mg/dl, glucose: 15 mg/dl, and opening pressure: 18 cmH_2_O. The EMG-NCV was conducted and acute axonal motor neuropathy (AMAN) variant of GBS was diagnosed. The motor nerve conduction and F-wave response were measured on the median, ulnar, tibial, and peroneal nerves. The sensory nerve conduction was measured using median, ulnar, and sural nerves. The normal NCV without evidence of conduction block, and normal sensory nerve action potential (SNAP) amplitude along with reduced compound muscle action potential (CMAP) amplitude were found in both upper and lower extremities. The electromyographic findings were reduced voluntary recruitment and increased insertional activity. The patient was treated with five consequent days of IVIG but the disease progressed and she developed dyspnea and was intubated. Arrhythmia due to autonomic dysfunction was also developed and pacemaker was inserted. The IVIG was used for further 7 days. The symptoms improved gradually and the patient was discharged after 42 days of hospitalizations with improvements in forces of lower limbs (4 / 5).

## Discussion

The COVID-19 is the third outbreak of coronaviruses after SARS and MERS, which occurred in 2002 and 2012; respectively. The SARS and MERS diseases led to less than 2000 deaths but the current pandemic outbreak of COVID-19 has claimed more than one million lives in less than a year. All three viruses were found to involve vital organ systems including nervous system [22–24]. The incidence of neurological manifestations was assessed in our study among hospitalized individuals with positive RT-PCR of SARS-CoV-2. The smell and taste dysfunctions were the third most common symptoms in our cases after fever and cough. It was identified that about two-third of our participants experienced smell and taste dysfunctions that resolved partially or completely in 98% of cases after 16 weeks of discharge from hospital. Myalgia (25%), headaches (13%), dizziness (12%), and cerebrovascular events (1%) were also reported in participants. Three individuals presented status epilepticus, post-infectious demyelinating myelitis, and AMAN variant of GBS.

The involvement of nervous system in COVID-19 disease can be attributed to the direct invasion of virus or secondary para-infectious mechanisms. The SARS-CoV-2 was found to enter the human cells using angiotensin converting enzyme 2 (ACE2) receptors [25] that can be expressed on nasal goblet and ciliated epithelial cells as well as oligodendrocytes [26, 27]. The detection of the SARS-CoV-2 in the CSF of cases with encephalopathy [28, 29] and demyelinating disorder [30] was an important clue to show that the virus could be neuroinvasive. Furthermore, viral particles were found in the brain tissue of cases with the COVID-19 disease [31, 32]. It should be noted that these results should be interpreted with caution [33]. Small numbers of cases were identified and some studies reported no evidence of the SARS-CoV-2 detection in the CSF or brain tissue of confirmed COVID-19 cases with neurological symptoms [34, 35]. The hyper-inflammatory state due to over-production of cytokines (e.g. interleukin-6 and tumor necrosis factor) also known as “cytokine storm” was reported to be another plausible underlying mechanism of neuronal damage in COVID-19 disease [36].

Several studies reported that hyposmia/ anosmia and dysgeusia were about 20 to 98% prevalent among confirmed cases with COVID-19 [7, 8, 37–39]. The discrepancies in results can be due to the methodological differences. The study design, sample size, recruitment setting (inpatients vs. outpatients), and model of testing smell sensation (self-reporting survey vs. physical exam) could significantly affect the results. It was found that anosmia was more common in non-hospitalized individuals than hospitalized ones with COVID-19 [40, 41]. Our study showed that loss of smell and taste was associated with reduced odds of severe cases and death due among hospitalized cases. A recent study on 576 inpatients also demonstrated that cases with anosmia had lower mortality rate and less severe course of the disease [42]. The follow-up of individuals with the COVID-19 and hyposmia/ anosmia was assessed in few studies. An observational cohort study reported that over 70% of cases with smell and taste dysfunctions had early improvements after 4 weeks of onset of anosmia [43]. The recovery in all cases was reported in another study in 28 days [44]. A 6-month follow-up study on 434 individuals showed that over 70% had full or almost full recovery of loss of smell and about 2% of cases reported no improvements [45]. It was also reported that over 40% of participants developed parosmia with the median interval of 2.5 months after the onset of smell dysfunction [45]. Our study also showed that about 10% of cases with hyposmia/anosmia developed parosmia after 4 weeks of discharge that increased to over 30% after 8 weeks. Longer follow-up periods are needed to assess the prognosis of altered smell functions.

A systematic literature review estimated that myalgia (28.5%) and headaches (14%) were common manifestations of COVID-19 [46]. In consistent with this study, it was previously found that headaches were independent predictors of lower risk of mortality among hospitalized cases [47]. We found that migraine-like episodes were the most frequent phenotype of headaches among the participants. A recent study also showed that headaches were common manifestations in COVID-19 and they were mostly presented as bilateral severe headaches at the time of admission with migraine phenotype [48] Individuals who diagnosed with COVID-19 were found to be at 0.5 to 1.3% risk of subsequent cerebrovascular events including ischemic stroke, ICH, and cerebral venous thrombosis [10, 49]. It was reported that among individuals with COVID-19 and stroke, older age, higher baseline NIHSS, and cryptogenic stroke were associated with early mortality [50].

To date, few cases with post-infectious demyelinating conditions of the central nervous system (CNS) after the COVID-19 disease were diagnosed [13, 51]. The structure and replication of SARS-CoV-2 was reported to be similar with mouse hepatitis virus (MHV) [52]. The MHV was found to remain in mouse CNS after acute infection and could induce an immune-mediated, chronic demyelinating disease, similar to multiple sclerosis in humans [33, 53]. We found two individuals with the CNS demyelination in our medical centers (51, present study) during COVID-19 pandemic outbreak. Future longitudinal studies should assess the prevalence of these events in confirmed COVID-19 cases. The post-infectious demyelination of peripheral nervous system was also reported after recovery from COVID-19 disease. The GBS is a relatively rare disease with the incidence ranged from 0.81 to 1.89 per 100,000 person-years [54]. Of 873 hospitalized individuals in our study, one case developed AMAN variant of GBS. Another study with 1200 patients also reported the incidence of 0.4% for GBS [55]. Systematic review studies reported that the majority of cases with COVID-19 and GBS had acute inflammatory demyelinating polyradiculopolyneuropathy (AIDP) variant [56, 57]. The facial nerve palsy that was observed in our participant was also found in about half of the prior reported cases [56].

To our knowledge, this was the first multi-center prospective study assessed the course of different neurological manifestations associated with COVID-19 disease. The prospective design of the study could reduce the possible information bias. The study was also multi-center that can increase the external validity. There were some limitations to our study. Some symptoms including anosmia and dysgeusia are often under-reported by patients with severe disease and our results could be affected by this measurement bias. Participants were sampled from hospitals rather than community (hospital based vs. population based studies). This can lead to selection bias (Berkson’s bias); as many individuals with mild severity were not included in our study. A short period of follow-up and lacking control group were other major limitations that should be resolved in future studies. Some neurological symptoms (e.g. smell and taste sensations, headaches, and myalgia) were not objectively assessed and were reliant on individuals’ reports. Further studies should be conducted to assess the incidence of CNS demyelinating events and evaluate the course of altered smell sensation including parosmia and phantosmia.

## Conclusions

Neurological symptoms are prevalent among individuals with the COVID-19 and should not be under-estimated during the current pandemic outbreak. The smell and taste dysfunctions and headaches were common neurological symptoms that were associated with less severe cases and lower mortality rates.

## Data Availability

The datasets generated and/or analysed during the current study are not publicly available but are available from the corresponding author on reasonable request.

## Abbreviations

AQP-4: Aquaporin-4 receptor
AIDP: Acute inflammatory demyelinating polyradiculopolyneuropathy
AMAN: Acute axonal motor neuropathy
CI: Confidence interval
CMAP: Compound muscle action potential
COVID-19: Coronavirus disease 2019
CRP: C-reactive protein
CSF: Cerebrospinal fluid
CT: Computed tomography
EEG: Electroencephalography
EMG-NCV: Electromyography and nerve conduction velocity
GBS: Guillain-Barré syndrome
ICH: Intracerebral hemorrhage
IVIG: Intravenous immunoglobulin
LDH: Lactate dehydrogenase
MERS: Middle East respiratory syndrome
MOG: Myelin oligodendrocyte glycoprotein
MRI: Magnetic resonance imaging
NIHSS: National Institute of Health stroke scale
NMDA: N-methyl-D-aspartate
OR: Odds ratio
RT-PCR: Reverse-transcription polymerase chain reaction
SARS-CoV: Severe acute respiratory syndrome coronavirus
SD: Standard deviation
SNAP: Sensory nerve action potential
WHO: World Health Organization
